# Novel Therapeutic Targets for Fibrodysplasia Ossificans Progressiva: Emerging Strategies and Future Directions

**DOI:** 10.7759/cureus.42614

**Published:** 2023-07-28

**Authors:** Usman Shaikh, Anoosha Khan, Priya Kumari, Anzal Ishfaq, Chukwuyem Ekhator, Paras Yousuf, Raghu Halappa Nagaraj, Hassan Raza, Ubaid Ur Rehman, Mohammad Uzair Zaman, Gautham Lakshmipriya Vetrivendan, Nhan Nguyen, Bijan Kadel, Tenzin N Sherpa, Ashraf Ullah, Sophia B Bellegarde

**Affiliations:** 1 Internal Medicine, Dow University of Health Sciences, Karachi, PAK; 2 Medicine, Jinnah Postgraduate Medical Centre, Karachi, PAK; 3 Internal Medicine, Mayo Hospital, Lahore, PAK; 4 Neuro-Oncology, New York Institute of Technology, College of Osteopathic Medicine, Old Westbury, USA; 5 Emergency Medicine, Jinnah Postgraduate Medical Centre, Karachi, PAK; 6 Surgery, Avalon University School of Medicine, Willemstad, CUW; 7 Internal Medicine, Lahore Medical and Dental College, Lahore, PAK; 8 Medicine, Bacha Khan Medical College, Mardan, PAK; 9 Medicine, Karpaga Vinayaga Institute of Medical Sciences and Research Centre, Maduranthakam, IND; 10 Medicine, University of Debrecen, Debrecen, HUN; 11 Internal Medicine, Nepal Medical College and Teaching Hospitals, Kathmandu, NPL; 12 Pathology and Laboratory Medicine, American University of Antigua, Saint John's, ATG

**Keywords:** crispr-cas9, mutant acvr1 gene, review, trauma, dysplasia, stoneman syndrome, fibrodysplasia ossificans progressiva

## Abstract

Fibrodysplasia ossificans progressiva (FOP), also known as Stoneman syndrome, is a rare genetic disorder characterized by abnormal bone development caused by activating mutations of the *ACVR1 *gene. FOP affects both the developmental and postnatal stages, resulting in musculoskeletal abnormalities and heterotopic ossification. Current treatment options for FOP are limited, emphasizing the need for innovative therapeutic approaches. Challenges in the development of management criteria for FOP include difficulties in recruitment due to the rarity of FOP, disease variability, the absence of reliable biomarkers, and ethical considerations regarding placebo-controlled trials. This narrative review provides an overview of the disease and explores emerging strategies for FOP treatment. Gene therapy, particularly the CRISPR-Cas9 (clustered regularly interspaced short palindromic repeats-associated protein 9) system, holds promise in treating FOP by specifically targeting the *ACVR1* gene mutation. Another gene therapy approach being investigated is RNA interference, which aims to silence the mutant *ACVR1 *gene. Small molecule inhibitors targeting glycogen synthase kinase-3β and modulation of the bone morphogenetic protein signaling pathway are also being explored as potential therapies for FOP. Stem cell-based approaches, such as mesenchymal stem cells and induced pluripotent stem cells, show potential in tissue regeneration and inhibiting abnormal bone formation in FOP. Immunotherapy and nanoparticle delivery systems provide alternative avenues for FOP treatment.

## Introduction and background

Fibrodysplasia ossificans progressiva (FOP), also known as Stoneman syndrome, is an extremely rare genetic disorder characterized by abnormal bone development. The condition is caused by activating mutations of Activin receptor A type I (ACVR1), a receptor responsible for bone morphogenetic proteins [1]. FOP affects both the developmental process and postnatal aspects, with congenital bilateral hallux valgus deformities being a hallmark of this condition [2]. The musculoskeletal characteristics of FOP are linked to dysregulated chondrogenesis, and the condition is characterized by heterotopic ossification (HO), which can occur spontaneously or as a result of trauma [3]. Flare-ups and painful soft tissue swelling often precede localized HO [4]. Initially, ossification mainly affects regions near the axial skeleton before progressing to the appendicular skeleton and other areas [5]. This abnormal bone formation restricts joint mobility and movement, with additional complications such as speech and swallowing difficulties, airway restriction, and impaired breathing [6].

To advance the understanding and treatment of FOP, it is crucial to conduct research on innovative therapeutic targets. This narrative review aims to develop disease-modifying medications, improve symptom management, and halt the excessive ossification process. By comprehending the molecular mechanisms and signaling pathways involved in bone formation, specific intervention targets can be identified, leading to the creation of targeted drugs that can slow down or stop excessive ossification [7]. The development of more effective pain relief and anti-inflammatory treatments will enhance the overall quality of life for individuals with FOP. Furthermore, uncovering therapeutic approaches that specifically target the underlying mechanisms of the disease may result in more efficient therapies with fewer side effects [8]. This article also offers insights into normal bone development and regeneration, enabling the development of personalized medical strategies that improve treatment effectiveness while minimizing side effects [9]. This review aims to provide a comprehensive overview of current knowledge, assess novel strategies, and guide future research endeavors toward developing effective treatments for FOP. It discusses the limitations of current treatment options for FOP, the challenges in identifying novel therapeutic targets, and emerging strategies such as gene therapy, small molecule approaches, stem cell-based approaches, and other innovative methods.

## Review

Epidemiology

FOP is a rare hereditary disorder characterized by congenital abnormalities in the great toes, thumbs, and vertebrae, as well as the progressive formation of bone in skeletal muscles [10]. There is no specific racial, ethnic, sex, or geographic predisposition, although cases have been documented across various groups [11]. The frequency of FOP is estimated to be one in 100,000 based on studies conducted in French and British populations. Regional variations in prevalence exist, with higher rates observed in certain areas. Prevalence rates range from 0.65 to 0.47 per million in North America and Western Europe, to 0.27 per million in Latin America, and lower rates in Africa and the Asia-Pacific region. The commonly cited estimated prevalence of FOP is 0.5 per million individuals, except in the United Kingdom (UK) and France where it exceeds 0.5 per million [12].

Clinical presentation

FOP is characterized by several distinct symptoms and manifestations. At birth, individuals with FOP often exhibit bilateral hallux valgus. One of the defining features of FOP is the occurrence of recurrent and painful soft tissue swellings, known as flare-ups. These flare-ups are triggered by gradual HO, particularly in response to soft tissue injuries such as immunizations or surgeries [13]. In infants, scalp nodules may be the initial symptom. FOP can also lead to limb abnormalities, specifically affecting the fingers. Thumb malformations, caused by either hypoplasia or dysplasia, are commonly observed. Scoliosis is prevalent in FOP and can worsen rapidly due to paravertebral lesions, exacerbating thoracic insufficiency syndrome [3,9,14]. Developmental hip dysplasia affects approximately 60% of individuals experiencing acute hip pain. Pelvic radiographs often reveal congenitally short and wide femoral necks. Osteochondromas are present in up to 90% of cases, with the proximal medial tibia being the most commonly affected. Additionally, approximately 50% of FOP cases experience conductive hearing loss, which tends to worsen gradually over time [14]. The combination of immobilization and accelerated bone turnover in FOP also increases the risk of developing renal stone disease. Flare-ups of lymphedema are also observed in some cases [13].

Genetics

FOP is a monogenic condition primarily associated with a mutation in the *ACVR1/ALK2* gene, located on chromosome 2, which plays a role in the bone morphogenetic protein (BMP) signaling system. This gene produces the ALK2 receptor, a transmembrane serine/threonine kinase receptor that interacts with BMPs present in the bone matrix [15,16]. The presence of BMPs triggers the formation of heterotopic bone in skeletal muscles. In the majority of cases with FOP, a nucleotide change occurs in the *ACVR1/ALK2 *gene at codon position 617, where guanine is replaced by adenine. This mutation leads to a substitution in the ALK2 protein, with histidine replacing arginine at codon position 206 [17].

Therapeutic approaches

Currently, there is no effective medical treatment for HO in FOP. The main approach to management is avoiding muscular injury or trauma. Surgery to remove ossifications often leads to recurrence and growth. Bracing is ineffective for spinal malformations, but limiting activities can help reduce trauma. Consultations on occupational therapy and career education are helpful [18]. High-dose corticosteroids can manage flare-up symptoms temporarily, and other medications like non-steroidal anti-inflammatory drugs (NSAIDs), cyclooxygenase 2 (COX2) inhibitors, mast cell inhibitors, and muscle relaxants may be used to treat subsequent flare-ups. Inhibiting the *ACVR1/ALK2* gene-related pathway, which prevents abnormal bone formation, is being explored as a potential therapy. Clinical studies are underway for medications such as palovarotene and rapamycin, and surgery is typically not a preferred treatment option for FOP patients [19].

Numerous medications, including palovarotene and rapamycin, are currently undergoing clinical trials for the treatment of Stoneman syndrome. However, these medications have certain limitations and restrictions for some individuals with FOP who also have concurrent conditions. For instance, palovarotene, a recognized teratogen, can result in limb deformities in the developing fetus and may have negative effects on growth plates, hearing, and eyesight in children. While humans generally tolerate the medication well, concerns primarily revolve around mucocutaneous adverse effects. On the other hand, rapamycin use in kidney transplant patients may pose risks such as proteinuria, dyslipidemia, and oral ulcers [20]. Patients with vascular abnormalities treated with rapamycin have experienced toxicity in the blood, bone marrow, and digestive system. A black-box warning exists for rapamycin, cautioning against its use in patients with liver conditions due to an increased risk of infection and immunosuppression [21]. These challenges contribute to the complexity of developing therapeutics for FOP.

The development of novel therapeutic targets for FOP faces several obstacles. The rarity and heterogeneity of the disease, limited understanding of its underlying mechanisms, lack of suitable animal models, and concerns regarding safety and the risk of flare-ups all contribute to these challenges [22]. FOP is characterized by an exaggerated inflammatory response, and any tissue injury or trauma can trigger flare-ups and subsequent formation of abnormal bone. The limited understanding of the disease hinders the identification of precise targets for therapeutic intervention, and accurate animal models that faithfully replicate FOP characteristics are scarce [23]. The risk of exacerbating the condition poses difficulties in implementing therapeutic interventions such as surgery or drug treatments [24].

The exploration of novel therapeutic targets for FOP holds promise in modifying the disease, improving symptom management, tailoring treatment approaches, reducing side effects, and advancing our knowledge of bone biology. These potential advantages offer hope for individuals living with the challenging condition of FOP, providing opportunities for advanced symptom management, precision, and personalized medicine, and minimized side effects.

Gene therapy

CRISPR-Cas9

Gene therapy, specifically the CRISPR-Cas9 (clustered regularly interspaced short palindromic repeats-associated protein 9) system, is a potential approach for treating FOP. With the aid of the ground-breaking gene-editing tool CRISPR-Cas9, researchers may accurately alter a gene's DNA sequence [25]. Researchers are looking at the prospect of fixing the mutation in the *ACVR1* gene, which causes FOP, in the context of the disease. By editing the *ACVR1* gene with CRISPR-Cas9, researchers hope to stop the aberrant bone growth associated with FOP [26]. A guide RNA molecule that matches the target DNA sequence and the Cas9 enzyme, which chops the DNA at the target spot, are the two components of the CRISPR-Cas9 system. Using a template DNA that has the necessary correction, the cell then repairs the damaged DNA. This way, CRISPR-Cas9 can introduce specific changes in the genome of living cells [27,28]. 

RNA Interference (RNAi)

Another gene therapy strategy being researched for FOP is RNAi. RNAi is a normal biological process that controls gene expression by silencing particular target genes. Researchers are looking at using RNAi to prevent the expression of the mutant *ACVR1 *gene in the case of FOP. It could be able to stop the abnormal bone growth found in FOP by lowering the synthesis of the mutant ACVR1 protein [29]. Small RNA molecules that attach to complementary mRNA molecules and stop them from being translated into proteins are the basis of RNAi. There are several ways to deliver these tiny RNA molecules to cells, including employing nanoparticles or viral vectors [30].

Adeno-associated Virus (AAV) Vectors

To deliver therapeutic genes to target cells during gene therapy, AAV vectors are frequently utilized. Scientists are investigating the possibility of using AAV vectors to transfer healthy copies of the *ACVR1* gene to FOP-affected cells. Researchers intend to stop the spread of FOP by delivering functioning *ACVR1* genes into these cells, which will return normal bone formation [31]. The virus that gave rise to AAV vectors is safe and can infect both dividing and non-dividing cells [32]. Most therapeutic genes can fit in a payload of up to 5 kilobases of DNA that AAV vectors can transport [33]. AAV vectors are superior to other viral vectors in a number of ways, including minimal immunogenicity, prolonged expression, and broad tissue tropism [34].

Receptor targeting

*Inhibition of Glycogen Synthase Kinase-3* (*GSK-3) β*

The enzyme GSK-3 is essential for several physiological functions, including bone development [35]. GSK-3 contributes to the promotion of aberrant bone development in FOP. GSK-3 small molecule inhibitors are being researched by researchers as potential treatments for FOP [36]. These inhibitors have demonstrated their capacity to lessen aberrant bone production in FOP models, yielding encouraging results in preclinical investigations [37]. Inhibitors of GSK-3, a downstream effector of the signaling pathway for BMP, function by preventing GSK-3 from acting. These substances can obstruct the osteogenic differentiation of cells and stop HO by inhibiting GSK-3 [38].

Modulation of BMP Signaling Pathway

For the formation and regeneration of bone, the BMP signaling pathway plays a crucial role. This pathway is responsible for coordinating the intricate processes involved in bone development and repair. However, in the case of FOP, the BMP signaling pathway malfunctions, leading to the abnormal formation of bone in soft tissues. In order to combat FOP and potentially delay its onset, researchers are actively exploring various small molecule substances that have the potential to modulate the BMP signaling pathway. These substances are being investigated with the aim of repairing the faulty pathway and halting the pathological process known as HO, where bone forms outside the skeletal system [2]. BMP antagonists, which are substances that bind to BMP receptors and prevent their activation. By blocking the interaction between BMPs and their receptors, these substances can help inhibit the aberrant signaling that triggers the formation of ectopic bone in FOP. This intervention acts as a roadblock, impeding the detrimental effects of the BMP signaling pathway gone awry [39,40].

*Targeting Peroxisome Proliferator-Activated Receptor *(*PPAR) γ*

A transcription factor called PPAR controls inflammation and adipocyte development. Targeting PPAR has been demonstrated to offer therapeutic promise for FOP in recent investigations. It has been discovered that activation of PPAR inhibits osteogenic differentiation of cells and lessens the development of HO [41]. Small molecule PPAR agonists are being investigated as a potential FOP therapeutic option. Agonists for PPAR function by attaching to PPAR and turning on its transcriptional activity. As a result, genes that encourage adipogenesis and by extension inhibit osteogenesis are expressed [42].

Stem cells

Mesenchymal Stem Cells (MSCs)

The ability to differentiate into numerous cell types, such as bone, cartilage, and adipose tissue, is possessed by MSCs, which may be used as a treatment for FOP, according to researchers. Scientists hope to encourage the regeneration of healthy tissue and stop the development of HO by administering MSCs to areas where abnormal bone production is occurring. Preclinical research has produced encouraging findings, showing that MSCs can inhibit bone growth in FOP models [43,44]. Bone marrow, adipose tissue, and umbilical cord blood are just a few of the sources from which MSCs can be obtained. MSCs may also undergo genetic modification to improve their therapeutic potency [9].

Induced Pluripotent Stem Cells (iPSCs)

Adult cells, like skin cells, can be reprogrammed to become pluripotent stem cells to create iPSCs, a specific form of stem cell. Any cell type in the body, including bone-forming cells, can be developed from iPSCs. In order to produce healthy bone-forming cells that can replace the abnormally formed bone, researchers are investigating the use of iPSCs in FOP. This strategy shows potential for regenerating healthy tissue and functionality [45,46]. A number of transcription factors, including Oct4, Sox2, Klf4, and c-Myc, can be added to somatic cells to produce iPSCs. Afterward, osteogenic cells derived from iPSCs can be created using particular culture conditions and growth agents [47,48].

Other approaches

Immunotherapy

An expanding field of study in FOP is immunotherapy, which uses the immune system to target particular cells or chemicals. Researchers are looking into several immunotherapeutic strategies that can specifically target the cells responsible for aberrant bone growth, such as monoclonal antibodies and immune checkpoint inhibitors. These treatments seek to prevent or lessen HO in FOP by regulating the immunological response [9,46]. Monoclonal antibodies are proteins that can attach to particular antigens on the surface of cells and cause the immune system to start destroying those cells. Immune checkpoint inhibitors are chemicals that can silence the immune system's signaling pathways, preventing it from attacking healthy cells. Researchers seek to improve the immune system's ability to recognize and destroy the abnormal cells that cause FOP by utilizing these immunotherapeutic agents [49,50].

Nanoparticle Delivery

Nanoparticles have the potential to deliver therapeutic medicines directly to the afflicted tissues in FOP. The use of nanoparticles to transport small medicines, gene-editing tools, and other therapeutic therapies selectively to regions of aberrant bone growth is being investigated by researchers. This customized delivery strategy attempts to improve the efficacy of prospective FOP therapies while reducing negative effects [51]. Nanoparticles are microscopic particles with specialized qualities such as size, shape, charge, and surface chemistry that may be manufactured. Nanoparticles can also be loaded with medicinal substances like medicines, genes, or proteins. Nanoparticles can be engineered to pass biological barriers like the blood-brain barrier or the skin and reach the desired regions. Nanoparticles can also be engineered to contain particular targeting ligands, such as antibodies or peptides, capable of recognizing and binding to specific receptors on target cells [52,53].

Current research on novel therapeutic targets

While the discovery of new treatment targets for FOP offers enormous potential, present research is mostly in the preclinical stage. Scientists are performing substantial research on the safety and efficacy of these developing techniques utilizing animal models and in vitro investigations. Several preclinical investigations on the molecular processes of HO and the impact of *ACVR1 *mutations have investigated possible treatment options for FOP. Among the promising targets are Activin A, BMPs, hypoxia, inflammation, and senescence. Activin A is a ligand that binds to ACVR1 and induces HO in FOP. In animal models of FOP, blocking Activin A signaling with monoclonal antibodies or small compounds has been demonstrated to decrease HO [54]. BMPs are ligands that bind to ACVR1 and regulate bone formation. In animal models of FOP, inhibiting BMP signaling with antibodies, small compounds, or gene therapy has been demonstrated to prevent or reverse HO [54]. Hypoxia is a low oxygen level state that triggers HO in FOP. In animal models of FOP, targeting hypoxia-inducible factors (HIFs) or downstream pathways with medicines or gene therapy has been demonstrated to reduce HO [6]. Inflammation is a response to tissue injury or infection that promotes HO in FOP. Modulating inflammatory cytokines, chemokines, or receptors with drugs or gene therapy has been shown to attenuate HO in animal models of FOP [6]. Senescence is a state of irreversible cell cycle arrest that contributes to HO in FOP. Eliminating senescent cells or inhibiting their secretory phenotype with drugs or gene therapy has been shown to reduce HO in animal models of FOP [55].

Clinical trials

There are currently six ongoing clinical trials for FOP registered on clinical trials.gov, all targeting Activin A signaling or BMP signaling (Table 1): (i) NCB000928: INCB000928 is a small molecule that inhibits activin A signaling by targeting the SMAD2/3 pathway. It is being tested in a phase 2 trial for efficacy, safety, and tolerability of INCB000928 versus placebo in patients with FOP; (ii) Garetosmab: Garetosmab is a human monoclonal antibody that binds to activin A and blocks its interaction with ACVR1. It is being tested in a phase 2 trial for the safety and efficacy of garetosmab versus placebo in adult patients with FOP; (iii) IPN60130: IPN60130 is another small molecule that inhibits activin A signaling by targeting the ALK4 receptor. It is being tested in a phase 2 trial for efficacy and safety of two dosage regimens of IPN60130 versus placebo in patients with FOP; (iv) KER-047: KER-047 is yet another small molecule that inhibits Activin A signaling by targeting the ALK4/5 receptors. It is being tested in a phase 2 trial for the safety and efficacy of KER-047 versus placebo in patients with FOP; (v) BCX9250: BCX9250 is a small molecule that inhibits BMP signaling by targeting the ALK2 receptor. It is being tested in a phase 1 trial for the safety, tolerability, pharmacokinetics, and pharmacodynamics of BCX9250 in patients with FOP; (vi) Palovarotene: Palovarotene is another small molecule that activates retinoic acid receptor gamma (RARγ), which inhibits activin A expression and signaling. It is being tested in a phase 3 trial for safety and efficacy in preventing new HO lesions in patients with FOP.

**Table 1:** Main features of ongoing clinical trials on novel therapeutic targets for FOP FOP: fibrodysplasia ossificans progressiva; HO: heterotopic ossification

ClinicalTrials.gov Identifier	Intervention	Population	Primary Outcome	Status
NCT05090891	INCB000928 + placebo	Adults and children with FOP	Proportion of participants who develop new HO lesions at 24 months	Not yet recruiting
NCT05394116	Garetosmab + placebo	Adults with FOP	Change in total volume of new HO lesions at 56 weeks	Recruiting
NCT05039515	IPN60130 + placebo	Adults and children with FOP	Change in total volume of new HO lesions at 24 months	Recruiting
NCT02745158	KER-047 + placebo	Adults with FOP	Change in total volume of new HO lesions at 24 weeks	Recruiting
NCT05027802	BCX9250 + placebo	Adults with FOP	Safety and tolerability of BCX9250	Recruiting
NCT04307953	Palovarotene + placebo	Adults and children with FOP	Proportion of participants who develop new HO lesions at 24 months	Recruiting

All trials are conducted in a randomized, double-blind, placebo-controlled manner across multiple centers. The trials of INCB000928, IPN60130, KER-047, and palovarotene consist of two arms: one arm receiving the intervention being tested and the other arm receiving a placebo. On the other hand, the garetosmab trial consists of three arms: one arm receiving a high dose of garetosmab, one arm receiving a low dose of garetosmab, and one arm receiving a placebo. The BCX9250 trial comprises six arms: five arms receiving different doses of BCX9250, and one arm receiving a placebo. In all these trials, whole-body low-dose computed tomography (CT) scans are utilized to measure the volume of new HO lesions.

Limitations and challenges

The results of current studies are not yet accessible since they are still being conducted or analyzed. However, various difficulties arise over the course of these experiments, including: (i) Recruitment: FOP is exceedingly uncommon. As a result, recruiting a sufficient number of qualified subjects for these studies might be difficult. The condition's rarity makes it difficult to find and recruit a substantial sample size, thereby affecting statistical power and trial outcomes representation; (ii) Variability: FOP exhibits significant heterogeneity, manifesting in varying degrees of disease severity, progression rates, and responses to treatment among affected individuals. This intrinsic diversity among the FOP patient group might complicate evaluating and generalizing study results. To achieve accurate and relevant findings, it is critical to account for these discrepancies; (iii) Biomarkers: Currently, reliable biomarkers for FOP that can predict disease activity, flare-ups, or response to treatment are lacking. The lack of such indicators complicates monitoring and optimizing intervention dose and timing during studies. Researchers are having difficulty analyzing the efficiency of the therapies being investigated since there are no exact indications of illness progression or therapy efficacy; (iv) Ethical considerations: FOP is a very disabling disease that causes permanent impairment and a worse quality of life. With no documented effective therapy or cure for FOP, the inclusion of a placebo group in the trials raises ethical concerns. Due to the tremendous influence of FOP on patients' well-being, withholding a potentially helpful intervention from individuals allocated to the placebo group raises ethical considerations. The ability to strike a balance between scientific rigor and ethical commitments becomes critical in these experiments.

Future directions and recommendations

One of the key future directions in the development of novel therapeutic targets for FOP is the continued identification and validation of targets. Advanced techniques such as genomics, proteomics, and high-throughput screening can aid in the identification of potential targets. By studying the genetic and protein profiles of individuals with FOP, researchers can identify specific molecules or pathways that are dysregulated in the disease. Validating these targets through in vitro and in vivo studies, as well as utilizing animal models that accurately recapitulate FOP characteristics, will be crucial in confirming their therapeutic potential.

As novel therapeutic targets for FOP are identified and experimental treatments are developed, rigorous safety and efficacy assessments are essential. Preclinical studies provide valuable insights into the potential risks and benefits of new treatments. These studies should encompass various aspects, including toxicity assessments, pharmacokinetics, and pharmacodynamics evaluations. Animal models that accurately represent FOP pathophysiology will be crucial in assessing the safety and efficacy of potential treatments. Moving forward, clinical trials will play a crucial role in evaluating the safety and efficacy of experimental therapies in human subjects. Rigorous trial designs, including randomized, double-blind, placebo-controlled studies, will be necessary to establish the effectiveness of new treatments. Long-term follow-up assessments will be vital to monitor treatment outcomes, including disease progression, quality of life, and adverse effects.

The successful translation of novel therapeutic targets into effective treatments for FOP requires the optimization of delivery methods. Gene therapy approaches, such as CRISPR-Cas9 and RNAi, rely on efficient and targeted delivery of therapeutic agents to the affected tissues. Finding effective ways to deliver these agents directly to the site of abnormal bone formation will be crucial for maximizing their therapeutic potential. Various delivery methods can be explored, including viral vectors, nanoparticles, and exosome-based approaches. Each method has its advantages and limitations in terms of efficiency, safety, and target specificity. Ongoing research should focus on optimizing these delivery methods to ensure effective and precise targeting of therapeutic agents to the affected tissues in FOP.

Given the complex nature of FOP, combination therapies may hold promise in maximizing treatment efficacy. Combining therapies that target different aspects of the disease can potentially provide synergistic effects and overcome individual treatment limitations. For example, combining gene therapy approaches with small molecule inhibitors or immunotherapies may offer a comprehensive and multi-faceted approach to addressing the diverse mechanisms underlying abnormal bone formation in FOP. To develop effective combination therapies, research efforts should focus on understanding the potential interactions, compatibility, and safety profiles of different treatment modalities. Preclinical studies and clinical trials can evaluate the efficacy and safety of combination therapies, paving the way for more comprehensive and effective treatment options for FOP.

## Conclusions

The advancement of new therapeutic targets for FOP offers great potential in enhancing symptom management, modifying the disease, and providing personalized treatment options for affected individuals. Emerging strategies, such as gene therapy, small molecules, stem cell-based approaches, immunotherapy, and nanoparticle delivery systems, are actively being investigated to target the underlying mechanisms of FOP and inhibit abnormal bone formation. Nonetheless, challenges such as recruitment difficulties due to the rarity of the disease, disease variability, lack of reliable biomarkers, and ethical considerations in placebo-controlled trials exist in developing novel therapeutic targets for FOP. However, ongoing research and clinical trials provide hope for improved management strategies and treatment options. Future directions include further identification and validation of targets, rigorous assessment of safety and efficacy, optimization of delivery methods, patient selection and stratification, and exploration of combination therapies. Through unraveling the complex mechanisms of FOP and developing targeted therapies, the goal of improved outcomes, disease modification, and ultimately finding a cure for FOP can be pursued.
